# Evaluation of a behavioural intervention to reduce perioperative midazolam administration to older adults

**DOI:** 10.1016/j.bjao.2023.100206

**Published:** 2023-07-11

**Authors:** Scott Seki, Molly Candon, Sushila Murthy, Gurmukh Sahota, Rachel R. Kelz, Mark D. Neuman

**Affiliations:** 1 Department of Anesthesiology and Critical Care, Perelman School of Medicine, University of Pennsylvania, Philadelphia, PA, USA; 2 Centers for Perioperative Outcomes Research and Transformation, PA, USA; 3 Department of Psychiatry, Perelman School of Medicine, The University of Pennsylvania, Philadelphia, PA, USA; 4 Department of Health Care Management, The Wharton School, The University of Pennsylvania, Philadelphia, PA, USA; 5 Leonard Davis Institute of Health Economics, University of Pennsylvania, Philadelphia, PA, USA; 6 Department of Surgery, Center for Surgery and Health Economics, Perelman School of Medicine, Philadelphia, PA, USA

**Keywords:** benzodiazepine, nudge, perioperative, quality

## Abstract

**Background:**

Older patients commonly receive benzodiazepines during anaesthesia despite guidelines recommending avoidance. Interventions to reduce perioperative benzodiazepine use are not well studied. We hypothesized an automated electronic medical record alert targeting anaesthesia providers would reduce administration of benzodiazepines to older adults undergoing general anaesthesia.

**Methods:**

We conducted a retrospective study of adults who underwent surgery at 5 hospitals within one US academic health system. One of the hospitals received an intervention consisting of provider education and an automated electronic medical record alert discouraging benzodiazepine administration to patients aged 70 years or older. We used difference-in-differences analysis to compare patterns of midazolam use 12-months before and after intervention at the intervention hospital, using the 4 non-intervention hospitals as contemporaneous comparators.

**Results:**

The primary analysis sample included 20,347 cases among patients aged 70 and older. At the intervention hospital, midazolam was administered in 454/4,240 (10.7%) cases pre-alert versus 250/3,750 (6.7%) post-alert (p<0.001). At comparator hospitals, respective rates were 3,186/6,366 (50.0%) versus 2,935/5,991 (49.0%) (p=0.24). After adjustment, the intervention was associated with a 3.2 percentage point (p.p.) reduction in the percentage of cases with midazolam administration (95% CI: (-5.2, -1.1); p=0.002). Midazolam dose was unaffected (adjusted mean difference -0.01 mg, 95% CI: (-0.20, 0.18); p=0.90). In 76,735 cases among patients aged 18–69, the percentage of cases with midazolam administration decreased by 6.9 p. p. (95% CI: (-8.0, -5.7); p<0.001).

**Conclusion:**

Provider-facing alerts in the intraoperative electronic medical record, coupled with education, can reduce midazolam administration to older patients presenting for surgery but may affect care of younger patients.

While research in the area of perioperative benzodiazepine administration to older surgical patients is ongoing,^1^^,^^2^ current guidelines recommend avoiding routine administration of benzodiazepines to older adults.^3^^,^^4^, ^5^, ^6^ Preoperative benzodiazepine administration before general anaesthesia does not improve patient satisfaction and may delay emergence.^7^ Despite this, a majority of older surgical patients in the US receive benzodiazepines during anaesthesia care.^8^^,^^9^

Few data exist as to how to discourage potentially avoidable perioperative benzodiazepine administration to older patients. One institution recently described a multi-component quality improvement initiative that decreased benzodiazepine administration to patients presenting for surgery.^10^^,^^11^ Further investigation into simple and scalable interventions focused on de-adoption of routine benzodiazepine administration may help clinicians, policy makers, and health systems optimize care of older surgical patients.

Nudges are environmental modifications designed to improve decision-making^12^^,^^13^; clinician-directed nudges, like automated electronic medical record (EMR) alerts, can promote guideline-concordant behaviours.^14^, ^15^, ^16^, ^17^, ^18^ The ability of nudges to discourage routine perioperative benzodiazepine administration to older surgical patients remains poorly characterized.

We tested the impact of an automated, clinician-directed EMR alert, coupled with educational outreach, on perioperative administration of midazolam to adults aged 70 years or older undergoing elective non-cardiac surgery. We hypothesized that exposure to the alert would be associated with decreased rates of benzodiazepine administration to patients requiring general anaesthesia.

## Methods

### Overview

This is a retrospective study of data from 5 hospitals in one academic US health system. One of the hospitals received a clinician-directed EMR alert discouraging benzodiazepine administration to surgical patients aged 70 years or older with educational outreach introducing the alert; the 4 remaining hospitals received no interventions and served as a grouped comparator. The effect of the alert on midazolam administration was estimated via difference-in-differences analysis (described below).^19^, ^20^, ^21^ The University of Pennsylvania Institutional Review Board exempted this study from review and waived the requirement for participant informed consent.

### Intervention

On April 4, 2019, a departmental conference focusing on potentially avoidable medications in older adults, such as benzodiazepines, was delivered to clinical anaesthesia staff at the intervention facility. This was immediately followed by a department-wide email summarizing the presentation. Selective administration and dose reduction were encouraged. On 17^th^ June, 2019, an automated EMR alert entitled “Delirium Prevention in Older Patients” was activated within the intraoperative anaesthesia record. The alert displays automatically for every anaesthesia provider who opens the intraoperative component of the anaesthesia record for patients aged 70 years or older. The alert text suggests avoidance of benzodiazepines with midazolam as the prototypical medication (Supplementary Figure 1). Also listed are other medication classes identified in American Geriatrics Society guidelines^3^ as being potentially inappropriate for use in older adults with a link to the guideline text. No similar interventions occurred at comparator hospitals over this period.

Planning of the intervention occurred separately from planning of this analysis. We focused our evaluation on benzodiazepines based on their frequency of use and evidence suggesting lack of benefit with potential for harm in most older surgical patients.^5^^,^^7^

### Participants and data sources

We obtained data on all patients aged 18 years or older who received general anaesthesia or sedation^22^ for elective non-cardiac surgical procedures between 18^th^ June, 2018 and 30^th^ June, 2020. For our main analysis, we defined the pre-alert period as including all cases performed between 18^th^ June, 2018 and 31^st^ May, 2019; we defined the post-alert period as including cases performed between 1^st^ July, 2019 and 30^th^ June, 2020. We excluded cases performed between 1^st^ June, 2019 and 30^th^ June, 2019 from the primary analysis to allow for a run-in period for alert implementation. The type of anaesthesia received was determined based on EMR documentation; we coded cases as general anaesthetics if they involved placement of a supraglottic airway or endotracheal tube. We excluded patients with ASA physical status classification^23^ V or VI, emergencies, and cases where regional anaesthetics were administered before induction of general anaesthesia.

### Outcomes

The primary outcome was a binary variable indicating the receipt of midazolam as recorded in the intraoperative anaesthesia record. We studied midazolam as non-midazolam benzodiazepines account for fewer than 1% of perioperative benzodiazepine administrations.^8^^,^^9^ The dose of midazolam administered (when given) was a secondary outcome.

### Independent variables

We obtained data from the EMR using Multicenter Perioperative Outcomes Group definitions^24^ on patient age, sex, race (Black, white, or other), Hispanic ethnicity, ASA physical status classification, body mass index, and 9 comorbidities (dementia, anxiety, cerebrovascular disease, cardiovascular disease, hypertension, chronic obstructive pulmonary disease, kidney disease, diabetes, peripheral vascular disease), use of inhaled anaesthetics, procedure duration, and surgical department.

### Statistical analysis

We performed a difference-in-differences (DID) analysis, a widely-used technique in economic research and policy evaluation. Our analytical plan was finalised in November 2020. Analysis began in May 2021. Unlike uncontrolled pre-post analyses, DID uses a contemporaneous comparison group (here, those hospitals that did not receive the intervention) to account for temporal changes in a particular outcome (here, midazolam use) that might otherwise confound causal inference (Supplementary Figure 2).^19^, ^20^, ^21^ A key assumption of DID is that trends in the outcome prior to the intervention are similar for both intervention and comparison groups (the so-called “parallel trends assumption”). Importantly, estimates from DID analysis are still valid even if the level of the outcome (e.g., the rate of midazolam use) differs for intervention versus control facilities, as long as trends in outcome prior to the intervention are parallel across treatment and control facilities.^19^, ^20^, ^21^

We used descriptive statistics and simple hypothesis tests to compare patient and procedure characteristics and outcomes at the intervention versus comparator hospitals. We fitted a linear probability model^25^^,^^26^ to predict our primary outcome based on the interaction of exposure group (intervention versus comparator hospital) and period (pre-versus post-alert), plus controls for patient characteristics, comorbidities, and surgical categories as above; the coefficient of this interaction term in the regression model corresponds to the impact of the intervention on the study outcome.^19^, ^20^, ^21^

We evaluated the parallel trends assumption via visual inspection and in a regression model estimating our outcome using data from the 12-month pre-intervention period only. This model predicted the primary study outcome along with an interaction between treatment arm (intervention versus comparator hospitals) and whether the case occurred during the first 6 months or the second 6 months of the pre-intervention period, plus all controls listed above. A non-significant estimate on the interaction term indicates parallel pre-intervention trends across the intervention versus control hospitals in the primary outcome.

To test the robustness of our findings, we repeated our main model to predict an endpoint of glycopyrrolate administration, a medication that was not specifically targeted by the alert. This served as a falsification test^19^, ^20^, ^21^; a finding of no effect on glycopyrrolate administration could be taken to confirm that our findings were likely to be due to the targeted impact of the EMR alert on midazolam versus some broader unspecified change in perioperative medication administration patterns. In contrast, finding that the alert increased or decreased glycopyrrolate use could be taken to imply the opposite. We also re-estimated our main model with an indicator variable for each month of study (time fixed-effects) and each hospital (group fixed-effects) to directly control for trends in the outcome variable over time and unobserved heterogeneity across hospitals. Finally, to assess whether inclusion of more than one case per patient may have affected our main results, we repeated the primary analysis at the patient-, rather than case-level, using the first listed case for each patient.

### Supplementary analyses

To assess whether the intervention affected younger patients, we fitted the same models in our secondary sample of patients aged 18–69 years who underwent general anaesthesia for elective non-cardiac surgery. We also analysed cases performed under sedation, since clinical decision-making for benzodiazepine use in this context may differ from that used for general anesthesia.^27^ To separate the potential effects of the components of the intervention (education vs EMR alert) on outcomes, we fitted a DID model that separately estimated changes in the outcome variable during the “education-only” phase of the intervention (i.e. between 4^th^ April, 2019 and 16^th^ June, 2019) and during the period after EMR alert activation (i.e. between 17^th^ June, 2019 and 30^th^ June, 2020) compared to the period before 4^th^ April, 2019.

### Data analyses and reporting

Analyses were performed using Stata 17.0 (Statacorp, College Station, TX). P<0.05 was our threshold for statistical significance. The strengthening the reporting of observational studies in epidemiology (STROBE) checklist appears in Supplementary Table 1.

## Results

### Characteristics of the primary study sample

After exclusions (Supplementary Table 2), our primary analysis included 20,347 cases performed in adults aged 70 or older (range 70–90 years old). 10,606 (52.1%) of these cases occurred in the 12 months before alert implementation, and 9,741 (47.9%) occurred after implementation. Over the full study period, most patient characteristics were similar at the intervention and comparator hospitals (Table 1), although cases at the intervention hospital were longer, involved patients with higher ASA physical status scores, and less frequently employed inhalational anaesthetics. Endocrine procedures were more common at the intervention hospital (12.6% of cases at intervention versus 1.6% of cases at comparator). Orthopaedic procedures were more common at the comparator hospitals (1.0% at intervention versus 23.8% at comparator). Other procedure types were similar between locations.

**Table 1:** Patient and procedure-level information (age 70 years and older).

	Control facilities (N=12,357)	Intervention Facility (N=7,990)	p-value
**Sex**			
Female	6,328 (51.2%)	3,661 (45.8%)	<0.001
Male	6,029 (48.8%)	4,329 (54.2%)	
Age	75.0 (72.0–80.0)	75.0 (72.0–79.0)	<0.001
**American Society of Anesthesiologists Physical Status Rating****^23^**			
1 (Healthy)	61 (0.5%)	16 (0.2%)	<0.001
2 (Mild systemic disease)	4,309 (34.9%)	2,069 (25.9%)	
3 (Severe systemic disease)	7,065 (57.2%)	5,492 (68.7%)	
4 (Severe systemic disease; constantly a threat to life)	922 (7.5%)	413 (5.2%)	
**Race**			
White	10,329 (83.6%)	6,617 (82.8%)	0.31
Black	1,744 (14.1%)	1,172 (14.7%)	
Other	284 (2.3%)	201 (2.5%)	
**Ethnicity**			
Non-Hispanic	12,176 (98.5%)	7903 (98.9%)	0.02
Hispanic	181 (1.5%)	87 (1.1%)	
**Comorbidities**			
Cerebrovascular disease	1,532 (12.4%)	1,074 (13.4%)	0.03
Dementia	178 (1.4%)	39 (0.5%)	<0.001
Anxiety	1,170 (9.5%)	694 (8.7%)	0.06
Cardiac disease	3,361 (27.2%)	2,146 (26.9%)	0.59
Hypertension	8,953 (72.5%)	5,881 (73.6%)	0.07
Chronic obstructive pulmonary disease	1,422 (11.5%)	1,138 (14.2%)	<0.001
Kidney disease	1,743 (14.1%)	1,414 (17.7%)	<0.001
Diabetes	3,383 (27.4%)	2,371 (29.7%)	<0.001
Peripheral artery disease	391 (3.2%)	270 (3.4%)	0.40
**Surgical Group**			
General Surgery	2,536 (20.5%)	1,118 (14.0%)	<0.001
Colorectal Surgery	206 (1.7%)	311 (3.9%)	
Endocrine Surgery	192 (1.6%)	1,004 (12.6%)	
Plastic Surgery	358 (2.9%)	285 (3.6%)	
Thoracic Surgery	273 (2.2%)	306 (3.8%)	
Transplant Surgery	0 (0.0%)	30 (0.4%)	
Trauma Surgery	38 (0.3%)	250 (3.1%)	
Urology	1,935 (15.7%)	1,274 (15.9%)	
Vascular Surgery	714 (5.8%)	420 (5.3%)	
Orthopaedic Surgery	2,936 (23.8%)	83 (1.0%)	
Gynaecological Surgery	533 (4.3%)	261 (3.3%)	
Neurosurgery	1,090 (8.8%)	843 (10.6%)	
Oral Maxillofacial, Ear Nose Throat, & Ophthalmologic Surgery	1,072 (8.7%)	980 (12.3%)	
Other	474 (3.8%)	825 (10.3%)	
Case duration (min)	120 (76–191)	131 (80–212)	<0.001
**Maintenance anaesthesia type**			
Inhalational anaesthetic	11671 (94.5%)	6,075 (76.0%)	<0.001
Total intravenous anaesthesia	686 (5.6%)	1,915 (24.0%)	

### Midazolam administration within the primary population

**Fig. 1:**
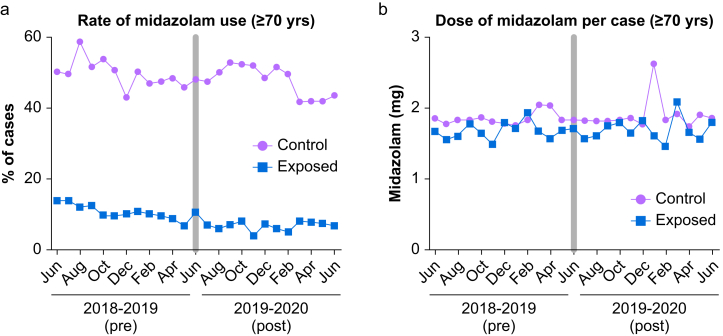
Monthly midazolam administration during study period for patients aged 70 years and older. Raw trends in perioperative midazolam use during study period in primary study population consisting of 20,347 cases). (A) Percentage of cases in which any midazolam was administered to patients. Each data point represents (number of cases using midazolam/total number of cases) calculated by month. (B) Average milligram dose of midazolam administered per case when used (n=6,825 of 20,347 cases). Each data point represents (mg sum of all midazolam administered/number of cases in which midazolam was administered) calculated by month. Control group represents aggregate data from unexposed hospitals (the comparator arm). Exposed group represents data from intervention site. Grey bar over June 2019 represents the implementation period.

In the period before alert deployment, at the intervention hospital, midazolam was administered in 10.7% (454/4,240) of cases involving patients 70 years or older compared with 6.7% (250/3,750) of cases post-alert (p<0.001). In contrast, at the comparator hospitals, midazolam was administered in 50.0% (3,186/6,366) of cases versus 49.0% (2,935/5,991) of cases respectively (p=0.24) (Figure 1). Supplementary Figure 3 shows patterns of midazolam administration by month across individual study hospitals.

For cases at the intervention hospital, the mean midazolam dose per case was 1.69 mg (SD 0.68 mg) pre-alert versus 1.72 mg (SD 0.73 mg) post-alert (p=0.59). At comparator facilities, the mean dose per case was 1.86 mg (SD 1.37 mg) versus 1.93 mg (SD 4.62 mg; p=0.47) respectively (Figure 1). After adjustment for patient and procedure-level factors, exposure to the alert was associated with a significant decrease in the percentage of cases in which midazolam was administered (difference-in-differences (DID) estimate -3.2 percentage points (p.p.); 95% CI (-5.2, -1.1); p=0.002; Table 2). The midazolam dose administered did not change (DID estimate -0.01 mg, 95% CI (-0.20, 0.18 mg), p=0.90). We obtained similar findings in models that included hospital fixed effects and month fixed effects (Supplementary Table 3) and in a sample that used only the first available procedure for each patient (Supplementary Table 4, Supplementary Figure 4).

**Table 2:** Study outcomes among patients aged 70 years and older. Results obtained from linear regression models adjusted for patient age, sex, race, ethnicity, American Society of Anesthesiologists physical status classification, body mass index, comorbidities (dementia, anxiety, cerebrovascular disease, cardiovascular disease, hypertension, chronic obstructive pulmonary disease, kidney disease, diabetes, peripheral vascular disease), use of volatile general anaesthetics (e.g., sevoflurane, isoflurane), case duration, and surgical department performing procedure.^a^ Intervention effect is the difference-in-difference-estimate derived from the model. It is the coefficient of the interaction term between time period (pre versus post-intervention) and exposure included in the regression;^b^ Includes patients who received at least one dose of midazolam intraoperatively. CI (confidence interval); p.p. (percentage point)

Outcome	Control facilities (95% CI)		Exposed facility (95% CI)			
Percentage receiving any midazolam	Pre-intervention (N = 6,366)	Post-intervention (N = 5,991)	Pre-intervention (N = 4,240)	Post-intervention (N = 3,750)	Intervention effect (95% CI)^a^	P-value
	49.5% (48.4, 50.7%)	48.8% (47.7, 50.0%)	11.2% (10.2, 12.2%)	7.3% (6.4, 8.2%)	-3.2 p.p. (-5.2, -1.1)	0.002
Midazolam dose administered per case (mg)^b^	Pre-intervention (N = 3,186)	Post-intervention (N = 2,935)	Pre-intervention (N = 454)	Post-intervention (N = 250)	Intervention effect (95% CI)^a^	P-value
	1.86 (1.82, 1.91)	1.93 (1.76, 2.10)	1.66 (1.58, 1.74)	1.72 (1.62, 1.81)	-0.01 mg (-0.20, 0.18)	0.90

While we did note differences in the incidence of midazolam administration at intervention versus comparator facilities, we confirmed parallel trends over the pre-alert period by visual inspection and by regression analyses using data restricted to the pre-alert period only as above (DID estimate for differential trends at intervention vs comparator hospitals: 2.8 p. p.; 95% CI (-0.1, 5.7); p=0.06; Supplementary Table 5). Supplemental analysis did not show a change in the incidence of midazolam administration between the date of the department educational session and the date of EMR alert activation compared to baseline (DID estimate 0.4 p. p.; 95% CI (-3.1, 3.9); p=0.831); midazolam administration was less frequent in the period after alert implementation compared to baseline (DID estimate -3.0 p. p.; 95% CI (-5.2, -0.9); p=0.005; Supplementary Table 6).

### Midazolam administration outcomes outside primary population

**Fig. 2:**
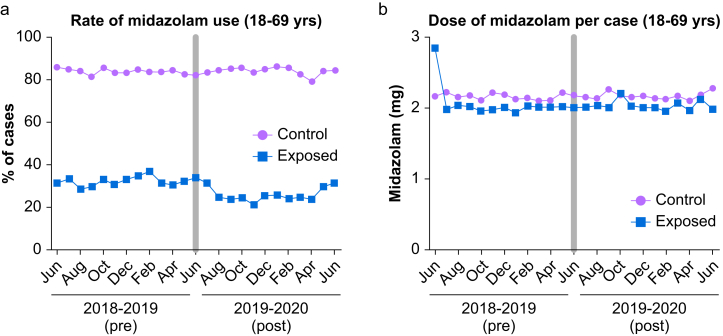
Monthly midazolam administration during study period for patients age 18 through 69 years old. Raw trends in perioperative midazolam use during study period in cases involving patients outside the age-range targeted by the alert (n=76,735 total cases). (A) Percentage of cases in which any midazolam was administered to patients. Each data point represents (number of cases using midazolam/total number of cases) calculated by month. (B) Average milligram dose of midazolam administered per case when prescribed (n=47,908 of 76,735 cases). Each data point represents (mg sum of all midazolam administered/number of cases in which midazolam was administered) calculated by month. Control group represents aggregate data from unexposed hospitals (the comparator arm). Exposed group represents data from intervention site. Grey bar over June 2019 represents the implementation period.

76,735 cases were performed on patients aged 18–69 years (Supplementary Table 7). At the intervention hospital, 32.1% (5,290/16,457) of cases involved midazolam administration pre-alert versus 25.9% (3,644/14,065) post-alert (p<0.001; Figure 2). At comparator hospitals, midazolam was administered in 84.1% (20,324/24,157) versus 84.6% of cases (18,650/22,056) pre-versus post-alert (p=0.21). After adjustment, exposure to the alert was associated with a statistically significant decrease in midazolam administration for cases involving patients aged 18–69 (DID estimate -6.9 p. p.; 95% CI (-8.0, -5.7); p<0.001; Table 3). The midazolam dose did not change (DID estimate -0.05 mg; 95% CI (-0.21, 0.10 mg); p=0.51; Table 3).

**Table 3:** Study outcomes among patients aged 18–69 years. Results obtained from linear regression models adjusted for patient age, sex, race, ethnicity, American Society of Anesthesiologists physical status classification, body mass index, comorbidities (dementia, anxiety, cerebrovascular disease, cardiovascular disease, hypertension, chronic obstructive pulmonary disease, kidney disease, diabetes, peripheral vascular disease), use of volatile general anaesthetics (e.g., sevoflurane, isoflurane), case duration, and surgical department performing procedure.^a^ Intervention effect is the difference-in-difference-estimate derived from the model. Itis the coefficient of the interaction term between time period (pre versus post-intervention) and exposure included in the regression;^b^ Includes patients who received at least one dose of midazolam intraoperatively. CI (confidence interval); p.p. (percentage point).

Outcome	Control facilities (95% CI)		Exposed facility (95% CI)			
Percentage receiving any midazolam	Pre-intervention (N = 24,157)	Post-intervention (N = 22,056)	Pre-intervention (N = 16,457)	Post-intervention (N = 14,065)	Intervention effect (95% CI)^a^	P-value
	83.0% (82.5, 83.5%)	83.8% (83.2, 84.3%)	33.6% (32.8, 34.3%)	27.5% (26.7, 28.2%)	-6.9 p.p. (-8.0, -5.7)	< 0.001
Midazolam dose administered per case (mg)^b^	Pre-intervention (N = 20,324)	Post-intervention (N = 18,650)	Pre-intervention (N = 5,290)	Post-intervention (N = 3,644)	Intervention effect (95% CI)^a^	P-value
	2.16 (2.13, 2.20)	2.17 (2.14, 2.21)	2.06 (1.96, 2.17)	2.02 (1.95, 2.09)	-0.05 (-0.21, 0.10)	0.505

21,718 cases with patients aged 70 years and older were performed under sedation rather than general anaesthesia (Supplementary Table 8,9). The percentage of sedation cases involving midazolam administration did not significantly change post-alert compared with pre-alert (DID estimate 1.0 p. p.; 95% CI (-0.4, 2.5); p=0.17; Supplementary Table 10). The midazolam dose also did not change (DID estimate -0.07 mg; 95% CI (-0.28, 0.14 mg); p=0.50; Supplementary Table 10).

### Sensitivity analyses

The alert did not significantly impact the percentage of cases with glycopyrrolate administration for patients aged 70 years or older (DID estimate -0.4 p. p.; 95% CI (-2.7, 2.0), p=0.75; Supplementary Table 11, Supplementary Figure 5A) or 18–69 years (DID estimate 0.4 p. p.; 95% CI (-0.9, 1.6), p=0.55; Supplementary Table 12, Supplementary Figure 5B).

## Discussion

Within one US academic health system, a clinician-facing, automated, EMR alert, preceded by clinician education, reduced the percentage of cases of general anaesthesia in which patients aged 70 years or older received midazolam by an adjusted 3.2 percentage points (p.p.). This finding was robust to adjustments for patient and procedure-level factors. The dose of midazolam, when administered, was not affected. Alert implementation was also associated with a decrease in the rate of midazolam administration for patients between 18 and 69 years undergoing general anaesthesia, despite the alert targeting older patients.

Notably, our intervention included both an educational component and an EMR alert, which were rolled out in sequence. We did not observe a change in midazolam administration in the period that immediately following the educational component, but during which the EMR alert had not yet been activated. This argues that impacts of education alone are unlikely to explain our findings. As we did not separately study an EMR alert at a site that did not receive departmental education, we cannot separate the impact of the alert alone versus the alert combined with a preceding educational intervention.

This study adds to prior knowledge of how to reduce perioperative midazolam administration to older adults. Previous studies in perioperative settings have shown EMR-based nudges, such as visual reminders embedded into the intraoperative chart, can improve compliance with guideline-directed patient management such as appropriate surgical antimicrobial prophylaxis.^14^^,^^28^ Donovan et al^11^ demonstrated a bundle of interventions, including identification of cognitively vulnerable older adults^10^ and implementation of default order-sets, can reduce administration of benzodiazepines though it is unclear which of their interventions drive this finding. We show that an EMR alert, preceded by clinician education, can reduce potentially discretionary perioperative midazolam administration.

There are multiple possible mechanisms that could explain the impact of our intervention on care patterns. As a visual cue, the alert may have served as a “priming” stimulus to encourage guideline-concordant care.^28^ Of note, the alert did not affect midazolam administration during cases performed under sedation where guidelines highlight its utility rather than dispensability.^27^ The alert, which is displayed as a delirium prevention effort, may have also added salience to decisions about midazolam administration by eliciting memories of patients with delirium.^13^ By establishing a norm of behaviour, the alert and the education session that preceded it may have drawn on established mechanisms for standardizing care practices.^29^, ^30^, ^31^ Overall, the alert may have influenced individual providers to administer midazolam less frequently, contributing to our observation of decreased midazolam administration beyond the targeted population. Other types of nudges previously shown to influence anaesthesia provider behaviours, such as changing default options^32^ and feedback tools^16^ were not employed in this initiative but represent potential areas for future study.

Our study has certain limitations. This quality improvement initiative took place at a major academic health system, and specifically at a hospital with relatively low rates of midazolam use. Baseline patterns of midazolam use at each hospital were not known during the design and implementation study phases and were only discovered during retrospective analysis. The generalizability of our findings to hospitals with different patterns of midazolam use remains to be determined. Reassuringly, the alert led to reduced rates of midazolam administration across surgical departments regardless of variable baseline rates of utilization. It is possible that our model failed to account for unobserved factors influencing midazolam usage patterns. For example, the final four months of our study occurred during the first wave of the COVID-19 pandemic, when case and patient mix varied considerably. Likewise, we did not consider variation amongst individual anaesthesia providers. These concerns are somewhat alleviated by the consistent results we obtained with analyses using time and group fixed effects to control for unobserved confounding. While we did not see a major impact of educational outreach in this study, education can powerfully alter provider behaviour, and a stronger outreach effort may have augmented observed effects.^33^ The link between perioperative administration of benzodiazepines and postoperative morbidity remains an area of active investigation,^1^^,^^2^ with recent studies reporting a single dose of preoperative midazolam to not significantly impact outcomes for older surgical patients.^34^^,^^35^ While our intervention was based on best available evidence, the science and guidelines surrounding this topic is continually evolving. This study was not designed to measure impact on patient outcomes. Future prospective work may consider assessing the end-impact of EMR alerts on outcomes attributable to perioperative medications such as midazolam.

Despite these limitations, our work is important for policy and practice. Among older surgical patients, receipt of potentially avoidable medications such as midazolam has been associated with adverse outcomes, including prolonged hospitalization, and guidelines recommend avoidance in this population.^3^^,^^5^^,^^6^ Nevertheless, midazolam is commonly administered to older patients presenting for surgery.^8^^,^^9^ Our findings suggest that an EMR alert, combined with education, can nudge anaesthesia providers away from administering midazolam to older adults perioperatively. Importantly, they also show that these alerts can substantially modify care beyond targeted populations.

In conclusion, an automated EMR alert directed toward anaesthesia providers and preceded by provider education and outreach, was associated with a decrease in perioperative midazolam administration to patients of all ages undergoing surgery. Further research is needed to determine the generalizability of findings and effects on patient outcomes. EMR nudges combined with education may be a useful strategy to reduce administration of benzodiazepines to older surgical patients.

## Details of authors’ contributions

All authors (SS, MC, SM, GS, RK, MN) made substantial contribution to conception and design, acquisition of data, or analysis and interpretation of data; drafting the article or revising it critically for important intellectual content; approve the version to be published; and agree to be accountable for all aspects of the work thereby ensuring that questions related to the accuracy or integrity of any part of the work are appropriately investigated and resolved.

## Funding

The 10.13039/100001080Patrick and Catherine Weldon Donaghue Medical Research Foundation, Grant Number 10084485 (M. Neuman). The funder had no role in the design and conduct of the study; collection, management, analysis, and interpretation of the data; preparation, review, or approval of the manuscript; and decision to submit the manuscript for publication.

## Declarations of interest

The authors declare that they have no conflicts of interest.

## References

[1] Spence J., Belley-Cote E., Jacobsohn E. (Jul 2020). Restricted versus liberal intraoperative benzodiazepine use in cardiac anaesthesia for reducing delirium (B-Free Pilot): a pilot, multicentre, randomised, cluster crossover trial. Br J Anaesth.

[2] Kowark A., Rossaint R., Keszei A.P. (Jul 15 2019). Impact of PReOperative Midazolam on OuTcome of Elderly patients (I-PROMOTE): study protocol for a multicentre randomised controlled trial. Trials.

[3] (Apr 2019). American Geriatrics society 2019 updated AGS beers criteria(R) for potentially inappropriate medication use in older adults. J Am Geriatr Soc.

[4] Mohanty S., Rosenthal R.A., Russell M.M., Neuman M.D., Ko C.Y., Esnaola N.F. (May 2016). Optimal perioperative management of the geriatric patient: a best practices guideline from the American College of surgeons NSQIP and the American Geriatrics society. J Am Coll Surg.

[5] Burfeind K.G., Zarnegarnia Y., Tekkali P., O'Glasser A.Y., Quinn J.F., Schenning K.J. (Aug 19 2022). Potentially inappropriate medication administration is associated with adverse postoperative outcomes in older surgical patients: a retrospective cohort study. Anesth Analg.

[6] Aldecoa C., Bettelli G., Bilotta F. (Apr 2017). European Society of Anaesthesiology evidence-based and consensus-based guideline on postoperative delirium. Eur J Anaesthesiol.

[7] Maurice-Szamburski A., Auquier P., Viarre-Oreal V. (Mar 3 2015). Effect of sedative premedication on patient experience after general anesthesia: a randomized clinical trial. JAMA.

[8] Lei V.J., Navathe A.S., Seki S.M., Neuman M.D. (Aug 2021). Perioperative benzodiazepine administration among older surgical patients. Br J Anaesth.

[9] Cozowicz C., Zhong H., Illescas A. (Mar 1 2022). The perioperative use of benzodiazepines for major orthopedic surgery in the United States. Anesth Analg.

[10] Whitlock E.L., Braehler M.R., Kaplan J.A. (Dec 2020). Derivation, validation, sustained performance, and clinical impact of an electronic medical record-based perioperative delirium risk stratification tool. Anesth Analg.

[11] Donovan A.L., Braehler M.R., Robinowitz D.L. (Dec 2020). An implementation-effectiveness study of a perioperative delirium prevention initiative for older adults. Anesth Analg.

[12] Patel M.S., Volpp K.G., Asch D.A. (Jan 18 2018). Nudge units to improve the delivery of health care. N Engl J Med.

[13] Thaler R.H.S.C.R. (2008).

[14] Hincker A., Ben Abdallah A., Avidan M., Candelario P., Helsten D. (Jul 2017). Electronic medical record interventions and recurrent perioperative antibiotic administration: a before-and-after study. Can J Anaesth.

[15] Tollinche L.E., Shi R., Hannum M. (Jul 2020). The impact of real-time clinical alerts on the compliance of anesthesia documentation: a retrospective observational study. Comput Methods Programs Biomed.

[16] O'Reilly-Shah V.N., Easton G.S., Jabaley C.S., Lynde G.C. (Dec 2018). Variable effectiveness of stepwise implementation of nudge-type interventions to improve provider compliance with intraoperative low tidal volume ventilation. BMJ Qual Saf.

[17] Kraus M.B., Poterack K.A., Strand N.H. (Oct 1 2021). Nudge theory in anesthesiology clinical practice. Int Anesthesiol Clin.

[18] Last B.S., Buttenheim A.M., Timon C.E., Mitra N., Beidas R.S. (Jul 12 2021). Systematic review of clinician-directed nudges in healthcare contexts. BMJ Open.

[19] Dimick J.B., Ryan A.M. (Dec 10 2014). Methods for evaluating changes in health care policy: the difference-in-differences approach. JAMA.

[20] Gertler P.J., Martinez S., Premand P., Rawlings L.B., Vermeersch C.M. (2016).

[21] Huntington-Klein N. (2021).

[22] (2023). Continuum of Depth of Sedation: Definition of general anesthesia and levels of sedation/analgesia.

[23] Hurwitz E.E., Simon M., Vinta S.R. (Apr 2017). Adding examples to the ASA-physical status classification improves correct assignment to patients. Anesthesiology.

[24] Sun E.C., Mello M.M., Vaughn M.T. (May 23 2022). Assessment of perioperative outcomes among surgeons who operated the night before. JAMA Intern Med.

[25] Huang F.L. (Jan 2 2022). Alternatives to logistic regression models in experimental studies. J Exp Educ.

[26] Liu Y., Colditz G.A., Kozower B.D. (Aug 1 2020). Association of medicaid expansion under the patient protection and affordable care act with non-small cell lung cancer survival. JAMA Oncol.

[27] (Mar 2018). Practice guidelines for moderate procedural sedation and analgesia 2018: a report by the American society of anesthesiologists task force on moderate procedural sedation and analgesia, the American association of oral and maxillofacial surgeons, American College of radiology, American dental association, American Society of Dentist Anesthesiologists, and Society of Interventional Radiology. Anesthesiology.

[28] Yoong S.L., Hall A., Stacey F. (Jul 1 2020). Nudge strategies to improve healthcare providers' implementation of evidence-based guidelines, policies and practices: a systematic review of trials included within Cochrane systematic reviews. Implement Sci.

[29] Cranney A., Lam M., Ruhland L. (Dec 2008). A multifaceted intervention to improve treatment of osteoporosis in postmenopausal women with wrist fractures: a cluster randomized trial. Osteoporos Int.

[30] Engers A.J., Wensing M., van Tulder M.W. (Mar 15 2005). Implementation of the Dutch low back pain guideline for general practitioners: a cluster randomized controlled trial. Spine (Phila Pa 1976).

[31] Feldstein A., Elmer P.J., Smith D.H. (Mar 2006). Electronic medical record reminder improves osteoporosis management after a fracture: a randomized, controlled trial. J Am Geriatr Soc.

[32] Ershoff B.D., Grogan T., Hong J.C., Chia P.A., Gabel E., Cannesson M. (May 2020). Hydromorphone unit dose affects intraoperative dosing: an observational study. Anesthesiology.

[33] Ostini R., Hegney D., Jackson C. (Mar 2009). Systematic review of interventions to improve prescribing. Ann Pharmacother.

[34] Kowark A., Berger M., Rossaint R., Schmid M., Coburn M., Group Po-S (Mar 1 2022). Association between benzodiazepine premedication and 30-day mortality rate: a propensity-score weighted analysis of the Peri-interventional Outcome Study in the Elderly (POSE). Eur J Anaesthesiol.

[35] Wang M.L., Min J., Sands L.P., Leung J.M., Perioperative Medicine Research G. (Sep 1 2021). Midazolam premedication immediately before surgery is not associated with early postoperative delirium. Anesth Analg.

