# Supplementary material for: Evaluation of a behavioural intervention to reduce perioperative midazolam administration to older adults
Source: BJA Open. 2023 Jul 11;7:100206. doi: 10.1016/j.bjao.2023.100206 (PMC10457488; doi:10.1016/j.bjao.2023.100206)
Supplement: Multimedia component 1 [file mmc1.docx]

**APPENDIX – Supplementary Data**

**Evaluation of a behavioral intervention to reduce perioperative midazolam administration to older adults**

Scott Seki MD, PhD,^a,b^ Molly Candon, PhD,^c,d,e^ Sushila Murthy MD, MPH,^a^ Gurmukh Sahota MD, PhD,^a^ Rachel R. Kelz MD, MSCE,^f^ Mark D. Neuman MD, MSc ^a,b,e,^

^a^ Department of Anesthesiology and Critical Care, Perelman School of Medicine, University of Pennsylvania, Philadelphia, PA.

^b^ Centers for Perioperative Outcomes Research and Transformation

^c^ Department of Psychiatry, Perelman School of Medicine, The University of Pennsylvania, Philadelphia.

^d^ Department of Health Care Management, The Wharton School, The University of Pennsylvania, Philadelphia

^e^ Leonard Davis Institute of Health Economics, University of Pennsylvania, Philadelphia, Pennsylvania

^f^ Department of Surgery, Center for Surgery and Health Economics, Perelman School of Medicine, Philadelphia, Pennsylvania.

**Corresponding Author Address:**

Mark D. Neuman, MD, MSc

Department of Anesthesiology and Critical Care

University of Pennsylvania Perelman School of Medicine

308 Blockley Hall

423 Guardian Drive

Philadelphia PA, 19106

neumanm@pennmedicine.upenn.edu

Tel: 215 746-7468

**Table of Contents**

|  | Page |
| --- | --- |
| Supplementary Figure 1: EMR alert design | 3 |
| Supplementary Figure 2: Difference-in-differences schematic | 4 |
| Supplementary Table 1: STROBE checklist for cohort studies | 5 |
| Supplementary Table 2: Reasons for case exclusion – age 70 years and older | 8 |
| Supplementary Figure 3: Raw monthly midazolam administration during study period for patients age 70 years and older at all facilities | 9 |
| Supplementary Table 3: Trends in midazolam administration using time and group fixed effects | 10 |
| Supplementary Figure 4: Monthly midazolam administration during study period for index cases involving patients age 70 years and older | 11 |
| Supplementary Table 4: Adjusted midazolam usage before and after alert implementation (age 70 years and older) – Index Cases | 12 |
| Supplementary Table 5: Rates of adjusted midazolam administration in pre-intervention period (age 70 years and older) | 13 |
| Supplementary Table 6: Isolating effect of education intervention on rate of midazolam administration to patients age 70 years and older | 14 |
| Supplementary Table 7: Demographics of patients age 18 through 69 years old | 15 |
| Supplementary Table 8: Reasons for case exclusion – age 70 years and older – cases under sedation | 17 |
| Supplementary Table 9: Patient and procedure-level information (age 70 years and older) – sedation cases | 18 |
| Supplementary Table 10: Study outcomes among patients aged 70 years and older – cases performed under sedation | 19 |
| Supplementary Figure 5: Monthly glycopyrrolate administration during study period | 20 |
| Supplementary Table 11: Adjusted glycopyrrolate administration rates before and after alert implementation (age 70 year and older) | 21 |
| Supplementary Table 12: Adjusted glycopyrrolate administration rates before and after alert implementation (age 18 through 69 years old) | 22 |


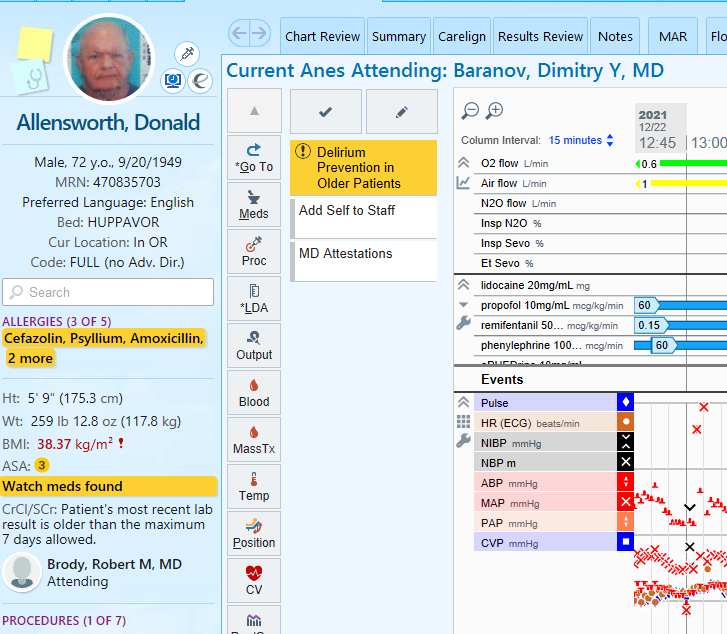

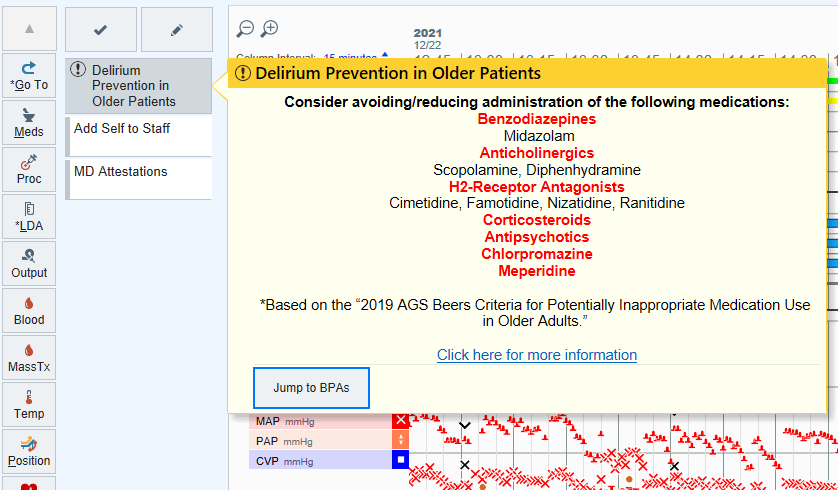


**Supplementary Figure 1: EMR alert design**

*Design of EMR alert notifying anesthesia providers of potential harms associated with medications identified by the 2019 American Geriatrics Society Beers Criteria. For all cases performed at the intervention site involving patients age 70 years and older, the alert appears in the upper left hand corner of the anesthesia provider’s intraoperative record. Inset: hovering over the yellow tab produces expanded alert window with links to further educational material. There are no required actions associated with the alert.*

**

**Supplementary Figure 2: Difference-in-differences schematic**

Difference-in-differences (DID) is an analytic method that utilizes regression to measure the treatment effect of an intervention on the treated relative to control. It does this by comparing the difference in an outcome before and after exposure to some intervention between a group that receives the intervention and a contemporaneous comparator. The left panel shows a representation of an uncontrolled pre-post study – without a comparator, the temporal trends preceding the intervention confound estimation of the treatment effect. In the right panel, inclusion of a control arm allows for temporal trends that exist independent of the intervention to be measured and accounted for. The DID estimate would be calculated as (C1-C0) – (E1-E0) or ΔC-ΔE (i.e. the difference in differences). If the pre-intervention trends in the outcome measure are similar between control and experimental groups, then performance of the DID calculation (ΔC-ΔE) cancels out pre-intervention trends to yield a post-intervention treatment effect on the treated.

**Supplementary Table 1:** STROBE Checklist of items for cohort studies

|  | Item No | Recommendation | Done? |
| --- | --- | --- | --- |
| **Title and abstract** | 1 | (*a*) Indicate the study’s design with a commonly used term in the title or the abstract | X |
|  |  | (*b*) Provide in the abstract an informative and balanced summary of what was done and what was found | X |
| Introduction | | |  |
| Background/rationale | 2 | Explain the scientific background and rationale for the investigation being reported | X |
| Objectives | 3 | State specific objectives, including any prespecified hypotheses | X |
| Methods | | |  |
| Study design | 4 | Present key elements of study design early in the paper | X |
| Setting | 5 | Describe the setting, locations, and relevant dates, including periods of recruitment, exposure, follow-up, and data collection | X |
| Participants | 6 | (*a*) Give the eligibility criteria, and the sources and methods of selection of participants. Describe methods of follow-up | X |
|  |  | (*b*) For matched studies, give matching criteria and number of exposed and unexposed | N/A |
| Variables | 7 | Clearly define all outcomes, exposures, predictors, potential confounders, and effect modifiers. Give diagnostic criteria, if applicable | X |
| Data sources/ measurement | 8* | For each variable of interest, give sources of data and details of methods of assessment (measurement). Describe comparability of assessment methods if there is more than one group | X |
| Bias | 9 | Describe any efforts to address potential sources of bias | X |
| Study size | 10 | Explain how the study size was arrived at | X |
| Quantitative variables | 11 | Explain how quantitative variables were handled in the analyses. If applicable, describe which groupings were chosen and why | X |
| Statistical methods | 12 | (*a*) Describe all statistical methods, including those used to control for confounding | X |
|  |  | (*b*) Describe any methods used to examine subgroups and interactions | X |
|  |  | (*c*) Explain how missing data were addressed | X |
|  |  | (*d*) If applicable, explain how loss to follow-up was addressed | N/A |
|  |  | (*e*) Describe any sensitivity analyses | X |
| Results | | |  |
| Participants | 13* | (a) Report numbers of individuals at each stage of study—eg numbers potentially eligible, examined for eligibility, confirmed eligible, included in the study, completing follow-up, and analysed | X |
|  |  | (b) Give reasons for non-participation at each stage | X |
|  |  | (c) Consider use of a flow diagram | X |
| Descriptive data | 14* | (a) Give characteristics of study participants (eg demographic, clinical, social) and information on exposures and potential confounders | X |
|  |  | (b) Indicate number of participants with missing data for each variable of interest | X |
|  |  | (c) Summarise follow-up time (eg, average and total amount) | X |
| Outcome data | 15* | Report numbers of outcome events or summary measures over time | X |
| Main results | 16 | (*a*) Give unadjusted estimates and, if applicable, confounder-adjusted estimates and their precision (eg, 95% confidence interval). Make clear which confounders were adjusted for and why they were included | X |
|  |  | (*b*) Report category boundaries when continuous variables were categorized | X |
|  |  | (*c*) If relevant, consider translating estimates of relative risk into absolute risk for a meaningful time period | N/A |
| Other analyses | 17 | Report other analyses done—eg analyses of subgroups and interactions, and sensitivity analyses | X |
| Discussion | | |  |
| Key results | 18 | Summarise key results with reference to study objectives | X |
| Limitations | 19 | Discuss limitations of the study, taking into account sources of potential bias or imprecision. Discuss both direction and magnitude of any potential bias | X |
| Interpretation | 20 | Give a cautious overall interpretation of results considering objectives, limitations, multiplicity of analyses, results from similar studies, and other relevant evidence | X |
| Generalisability | 21 | Discuss the generalisability (external validity) of the study results | X |
| Other information | | |  |
| Funding | 22 | Give the source of funding and the role of the funders for the present study and, if applicable, for the original study on which the present article is based | X |

*Give information separately for exposed and unexposed groups.

**Supplementary Table 2: Reasons for case exclusion – age 70 years and older**

| **Reason for case exclusion** | **Number excluded** |
| --- | --- |
| Emergencies | 1,535 |
| American Society of Anesthesiologists Physical Status rating 5 or 6 | 86 |
| Regional block performed | 698 |
| Cardiac surgery | 3,222 |
| Missing hospital location | 611 |
| Missing race | 1,462 |
| Missing case duration | 51 |
| Missing surgery department | 442 |
| Performed during implementation month | 1,132 |
| Total cases excluded* | 7,635 |
| Total included | 20,347 |

* Note: some cases excluded due to meeting more than one pre-specified exclusion criteria.

**Supplementary Figure 3: Raw monthly midazolam administration during study period for patients age 70 years and older at all facilities**

Raw trends in perioperative midazolam use during study period in primary study population consisting of 20,347 cases). Percentage of cases in which any midazolam was administered to patients. Each data point represents (number of cases using midazolam / total number of cases) calculated by month. Exposed group represents data from intervention site. Grey bar over June 2019 represents the alert implementation period.

**Supplementary Table 3: Trends in midazolam administration using time and group fixed effects**

|  | | **Model 1 (original)** | **Model 2 (time fixed effects)** | **Model 3 (time and group fixed effects)** |
| --- | --- | --- | --- | --- |
| *Variables accounted for in regression* | *Patient level factors* | X | X | X |
|  | *Procedure level factors* | X | X | X |
|  | *Time fixed effects* |  | X | X |
|  | *Group fixed effects* |  |  | X |
|  | | | | |
| Regression output | Intervention  effect ^a^ | -3.2 p.p. | -3.1 p.p. | -2.7 p.p. |
|  | 95% CI | (-5.2, -1.1) p.p. | (-5.1, -1.0) p.p. | (-4.6, -0.8) p.p. |
|  | p-value | p=0.002 | p=0.003 | p=0.006 |

Results obtained from linear regression models adjusted for patient age, sex, race, ethnicity, American Society of Anesthesiologists physical status classification, body mass index, comorbidities (dementia, anxiety, cerebrovascular disease, cardiovascular disease, hypertension, chronic obstructive pulmonary disease, kidney disease, diabetes, peripheral vascular disease),use of volatile general anesthetics (e.g., Sevoflurane, Isoflurane), case duration, and surgical department performing procedure. ^a^ Intervention effect is the difference-in-difference-estimate derived from the model. It is the coefficient of the interaction term between time period (12 months prior to intervention versus 12 months after) and exposure included in the regression; CI (confidence interval); p.p. (percentage point).

**A B**
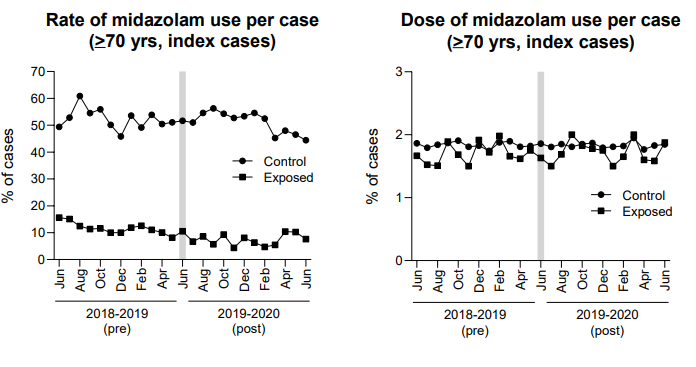


**Supplementary Figure 4: Monthly midazolam administration during study period for index cases involving patients age 70 years and older**

*Raw trends in perioperative midazolam use during study period in index cases (i.e. for patients who underwent multiple cases in the study period, only the first case is considered) involving patients age 70 years and older (n=10,913 cases). (A) Percentage of cases in which any midazolam was administered to patients. Each data point represents (number of cases using midazolam / total number of cases) calculated by month. (B) Average milligram dose of midazolam administered per case when prescribed (n=3,988 of 10,913 cases). Each data point represents (milligram sum of all midazolam administered / number of cases in which midazolam was administered) calculated by month. Control group represents aggregate data from unexposed hospitals (the comparator arm). Exposed group represents data from intervention site. Grey bar over June 2019 represents the implementation period.*

**Supplementary Table 4: Adjusted midazolam usage before and after alert implementation (patient age 70 years and older) – Index Cases**

| **Outcome** | **Control Facilities (95% CI)** | | **Exposed Facility (95% CI)** | |  |  |
| --- | --- | --- | --- | --- | --- | --- |
| Percentage receiving any midazolam | Pre-intervention  (N = 3,553) | Post-intervention  (N = 3,378) | Pre-intervention  (N = 2,171) | Post-intervention  (N = 1,811) | Intervention effect  (95% CI)^a^ | P-value |
|  | 52.8%  (51.3, 54.3%) | 51.4%  (49.9, 53.0%) | 11.8%  (10.4, 13.3%) | 6.5%  (5.2, 7.9%) | -3.9 p.p.  (-6.7, -1.1) | p=0.006 |
| Midazolam dose administered per case (mg)^b^ | Pre-intervention  (N = 1,866) | Post-intervention  (N = 1,750) | Pre-intervention  (N = 251) | Post-intervention  (N = 121) | Intervention effect  (95% CI)^a^ | P-value |
|  | 1.85  (1.82, 1.87) | 1.83  (1.81, 1.86) | 1.68  (1.61, 1.75) | 1.78  (1.66, 1.90) | 0.11  (-0.03, 0.26) | p=0.118 |

Results obtained from linear regression models adjusted for patient age, sex, race, ethnicity, American Society of Anesthesiologists physical status classification, body mass index, comorbidities (dementia, anxiety, cerebrovascular disease, cardiovascular disease, hypertension, chronic obstructive pulmonary disease, kidney disease, diabetes, peripheral vascular disease),use of volatile general anesthetics (e.g., Sevoflurane, Isoflurane), case duration, and surgical department performing procedure. For patients who underwent multiple procedures within the study window, only the first case (index case) is considered in the model. ^a^ Intervention effect is the difference-in-difference-estimate derived from the model. It is the coefficient of the interaction term between time period (pre versus post-intervention) and exposure included in the regression; ^b^ Includes patients who received at least one dose of midazolam intraoperatively. CI (confidence interval); p.p. (percentage points).

**Supplementary Table 5: Rates of adjusted midazolam administration in pre-intervention period (patient age 70 years and older)**

| **Outcome** | **Control Facilities (95% CI)** | | **Exposed Facility (95% CI)** | |  |  |
| --- | --- | --- | --- | --- | --- | --- |
| Percentage receiving any midazolam | Pre Period 1  (N = 3,438) | Pre Period 2  (N = 2,928) | Pre Period 1  (N = 2,358) | Pre Period 2  (N = 1,882) | Intervention effect  (95% CI)^a^ | P-value |
|  | 51.4%  (49.9, 53.0%) | 47.6%  (46.0, 49.3%) | 11.7%  (10.3, 13.0%) | 10.7%  (9.3, 12.2%) | 2.8 p.p.  (-0.1, 5.7) | 0.059 |

Results obtained from linear regression models adjusted for patient age, sex, race, ethnicity, American Society of Anesthesiologists physical status classification, body mass index, comorbidities (dementia, anxiety, cerebrovascular disease, cardiovascular disease, hypertension, chronic obstructive pulmonary disease, kidney disease, diabetes, peripheral vascular disease),use of volatile general anesthetics (e.g., Sevoflurane, Isoflurane), case duration, and surgical department performing procedure. ^a^ Intervention effect is the difference-in-difference-estimate derived from the model. It is the coefficient of the interaction term between time period (first six months prior to intervention versus second six months prior to intervention) and exposure included in the regression. CI (confidence interval); p.p. (percentage point).

**Supplementary Table 6: Isolating effect of education intervention on rate of midazolam administration to patients age 70 years and older**

| **Outcome** | **Control Facilities (95% CI)** | | | **Exposed Facility (95% CI)** | | | **Intervention Effect (95% CI) ^a^** | | | |
| --- | --- | --- | --- | --- | --- | --- | --- | --- | --- | --- |
| Percentage receiving any midazolam | Phase 0  N=5,362 | Phase 1  N=1,303 | Phase 2  N=6,204 | Phase 0  N=3,599 | Phase 1  N=841 | Phase 2  N=3,909 | Phase 0 vs Phase 1 | P-value  0 v 1 | Phase 0 vs Phase 2 | P-value  0 v 2 |
|  | 49.8%  (48.6, 51.0) | 48.0%  (45.5, 50.5) | 48.8%  (47.6, 49.9) | 11.5%  (10.4, 12.6) | 10.1%  (8.1, 12.0) | 7.4%  (6.5, 8.3) | 0.4 p.p.  (-3.1, 3.9) | 0.831 | -3.0 p.p.  (-5.2, -0.9) | 0.005 |
| Results obtained from linear regression models adjusted for patient age, sex, race, ethnicity, American Society of Anesthesiologists physical status classification, body mass index, comorbidities (dementia, anxiety, cerebrovascular disease, cardiovascular disease, hypertension, chronic obstructive pulmonary disease, kidney disease, diabetes, peripheral vascular disease),use of volatile general anesthetics (e.g., Sevoflurane, Isoflurane), case duration, and surgical department performing procedure. ^a^ Intervention effect is the difference-in-difference-estimate derived from the model. Itis the coefficient of the interaction term between time period (phase) and exposure included in the regression. CI (confidence interval); p.p. (percentage point). Phase 0 = prior to April 4, 2019. Phase 1 = education only phase from April 4, 2019 – June 18, 2019. Phase 3 = post-alert implementation phase = June 19, 2019 – June 30, 2020. | | | | | | | | | | |

**Supplementary Table 7: Demographics of patients age 18 through 69 years old**

|  | **Control Facilities (N=46,213)** | **Exposed Facilities**  **(N=30,522)** | **p-value** |
| --- | --- | --- | --- |
| **Sex** |  |  |  |
| Female | 26,792 (58.0%) | 18,083 (59.2%) | <0.001 |
| Male | 19,421 (42.0%) | 12,439 (40.8) |  |
| **Age** | 52.0 (38.0-61.0) | 54.0 (41.0-62.0) | <0.001 |
| **ASA** |  |  |  |
| 1 | 4,727 (10.2%) | 1,586 ( 5.2%) | <0.001 |
| 2 | 25,747 (55.7%) | 13,602 (44.6%) |  |
| 3 | 14,515 (31.4%) | 14,392 (47.2%) |  |
| 4 | 1,224 ( 2.7%) | 942 ( 3.1%) |  |
| **Race** |  |  |  |
| White | 33,198 (71.8%) | 21,970 (72.0%) | 0.47 |
| Black | 11,393 (24.7%) | 7,531 (24.7%) |  |
| Other | 1,622 ( 3.5%) | 1,021 ( 3.3%) |  |
| **Ethnicity** |  |  |  |
| Non-Hispanic | 44,053 (95.3%) | 29,504 (96.7%) | <0.001 |
| Hispanic | 2,160 ( 4.7%) | 1,018 ( 3.3%) |  |
| **Comorbidities** |  |  |  |
| Cerebrovascular disease | 2,332 ( 5.1%) | 2,321 ( 7.60%) | <0.001 |
| Dementia | 19 ( 0.0%) | 9 ( 0.0%) | 0.41 |
| Anxiety | 5,678 (12.3%) | 3,972 (13.0%) | 0.003 |
| CAD, MI or CHF | 3,880 ( 8.4%) | 2,783 ( 9.1%) | <0.001 |
| HTN | 16,834 (36.43%) | 12,436 (40.7%) | <0.001 |
| COPD | 1,706 ( 3.69%) | 2,039 ( 6.7%) | <0.001 |
| Kidney Disease | 2,474 ( 5.35%) | 2,784 ( 9.1%) | <0.001 |
| Diabetes | 7,456 (16.13%) | 6,743 (22.1%) | <0.001 |
| PAD | 448 ( 0.97%) | 366 ( 1.2%) | 0.002 |
| **Surgical Group** |  |  |  |
| Department of Surgery |  |  | <0.001 |
| General Surgery | 8,710 (18.9%) | 4,265 (14.0%) |  |
| Colorectal Surgery | 784 ( 1.7%) | 1,301 ( 4.3%) |  |
| Endocrine Surgery | 808 ( 1.8%) | 3,783 (12.4%) |  |
| Plastic Surgery | 3,406 ( 7.4%) | 2,597 ( 8.5%) |  |
| Thoracic Surgery | 393 ( 0.9%) | 715 ( 2.3%) |  |
| Transplant Surgery | 0 ( 0.0%) | 692 ( 2.3%) |  |
| Trauma Surgery | 298 ( 0.6%) | 1,009 ( 3.3%) |  |
| Urologic Surgery | 4,022 ( 8.7%) | 2,575 ( 8.4%) |  |
| Vascular Surgery | 1,185 ( 2.6%) | 618 ( 2.0%) |  |
| Orthopedic Surgery | 11,156 (24.1%) | 275 ( 0.9%) |  |
| Gynecologic Surgery | 5,844 (12.7%) | 3,184 (10.4%) |  |
| Neurosurgery | 2,851 ( 6.2%) | 2,849 ( 9.3%) |  |
| Oral Maxillofacial Surgery  Ear Nose Throat Surgery  Ophthalmologic Surgery | 5,170 (11.2%) | 4,409 (14.5%) |  |
| Other | 1,586 ( 3.4%) | 2,250 ( 7.4%) |  |
| **Case duration (min)** | 114.0 (74.0-182.0) | 134.0 (84.0-214.0) | <0.001 |
| **Maintenance anesthesia** |  |  |  |
| Inhalational | 43,742 (94.7%) | 23,481 (76.9%) | <0.001 |
| Total IV Anesthesia | 2,471 ( 5.3%) | 7,041 (23.1%) |  |

**Supplementary Table 8: Reasons for case exclusion – age 70 years and older – cases under sedation**

| **Reason for case exclusion** | **Number excluded** |
| --- | --- |
| Emergencies | 490 |
| American Society of Anesthesiologists Physical Status rating 5 or 6 or missing | 184 |
| Regional block performed | 1,066 |
| Cardiac surgery | 3,257 |
| Missing hospital location | 3,738 |
| Missing race | 1,414 |
| Missing case duration | 1,606 |
| Missing surgery department | 3,586 |
| Performed during implementation month | 1,477 |
| Total cases excluded* | 10,714 |
| Total included | 21,718 |

* Note: some cases excluded due to meeting more than one pre-specified exclusion criteria.

**Supplementary Table 9: Patient and procedure-level information (age 70 years and older) – sedation cases**

|  | **Control Facilities (N=15,214)** | **Intervention**  **Facility**  **(N=6,504)** | **p-value** |
| --- | --- | --- | --- |
| **Sex** |  |  |  |
| Female | 8,807 (57.9%) | 3,085 (47.4%) | <0.001 |
| Male | 6,407 (42.1%) | 3,419 (52.6%) |  |
| **Age** | 76 (72-80) | 74 (72-78) | <0.001 |
| **American Society of Anesthesiologists Physical Status Rating^18^** |  |  |  |
| 1 (Healthy) | 119 ( 0.78%) | 34 ( 0.52%) | <0.001 |
| 2 (Mild systemic disease) | 6,277 (41.3%) | 2,185 (33.6%) |  |
| 3 (Severe systemic disease) | 7,934 (52.2%) | 4,085 (62.8%) |  |
| 4 (Severe systemic disease; constantly a threat to life) | 884 ( 5.8%) | 200 ( 3.1%) |  |
| **Race** |  |  |  |
| White | 11,391 (74.9%) | 4,935 (75.9%) | 0.29 |
| Black | 3,321 (21.8%) | 1,362 (20.9%) |  |
| Other | 502 ( 3.3%) | 207 ( 3.2%) |  |
| **Ethnicity** |  |  |  |
| Non-Hispanic | 14,972 (98.4%) | 6,398 (98.4%) | 0.83 |
| Hispanic | 242 ( 1.6%) | 106 ( 1.6%) |  |
| **Comorbidities** |  |  |  |
| Cerebrovascular disease | 1,976 (13.0%) | 1,465 (22.5%) | <0.001 |
| Dementia | 147 ( 1.0%) | 17 ( 0.3%) | <0.001 |
| Anxiety | 1,472 ( 9.7%) | 561 ( 8.6%) | 0.015 |
| Cardiac disease | 3,879 (25.5%) | 1,664 (25.6%) | 0.89 |
| Hypertension | 11,007 (72.4%) | 4,653 (71.5%) | 0.22 |
| Chronic obstructive pulmonary disease | 1,604 (10.5%) | 804 (12.4%) | <0.001 |
| Kidney disease | 2,081 (13.7%) | 857 (13.2%) | 0.32 |
| Diabetes | 4,450 (29.3%) | 2,500 (38.4%) | <0.001 |
| Peripheral artery disease | 394 ( 2.6%) | 251 ( 3.9%) | <0.001 |
| **Surgical Group** |  |  |  |
| General Surgery | 7,300 (48.0%) | 5,786 (89.0%) | <0.001 |
| Colorectal Surgery | 138 ( 0.9%) | 45 ( 0.7%) |  |
| Endocrine and Oncologic Surgery | 88 ( 0.6%) | 41 ( 0.6%) |  |
| Plastic Surgery | 66 ( 0.4%) | 90 ( 1.4%) |  |
| Thoracic Surgery | 3 ( 0.0%) | 1 ( 0.0%) |  |
| Transplant Surgery | 0 ( 0.0%) | 4 ( 0.1%) |  |
| Trauma Surgery | 1 ( 0.0%) | 5 ( 0.1%) |  |
| Urologic Surgery | 723 ( 4.8%) | 135 ( 2.1%) |  |
| Vascular Surgery | 534 ( 3.5%) | 223 ( 3.4%) |  |
| Orthopedic Surgery | 2,862 (18.8%) | 3 ( 0.1%) |  |
| Gynecologic Surgery | 273 ( 1.8%) | 14 ( 0.2%) |  |
| Neurosurgery | 84 ( 0.6%) | 17 ( 0.3%) |  |
| Oral Maxillofacial, Ear Nose Throat, & Ophthalmologic Surgery | 2,286 (15.0%) | 52 (0.8%) |  |
| Other (Pain, Podiatry, Psych, Pulmonology, Radiation Oncology) | 856 ( 5.6%) | 88 (1.4%) |  |
| **Case duration (min)** | 44.0  (30.0-73.0) | 37.0  (27.0-53.0) | <0.001 |

**Supplementary Table 10: Study outcomes among patients aged 70 years and older – cases performed under sedation**

| **Outcome** | **Control Facilities (95% CI)** | | **Exposed Facility (95% CI)** | |  |  |
| --- | --- | --- | --- | --- | --- | --- |
| Percentage receiving any midazolam | Pre-intervention  (N = 7,625) | Post-intervention  (N = 7,589) | Pre-intervention  (N = 3,564) | Post-intervention  (N = 2,940) | Intervention effect  (95% CI)^a^ | P-value |
|  | 34.6%  (33.8, 35.5%) | 33.0%  (32.2, 33.8%) | 17.6%  (16.9, 18.3%) | 17.0%  (16.2, 17.7%) | 1.0 p.p.  (-0.4, 2.5) | 0.172 |
| Midazolam dose administered per case (mg)^b^ | Pre-intervention  (N = 3,217) | Post-intervention  (N = 2,885) | Pre-intervention  (N = 105) | Post-intervention  (N = 63) | Intervention effect  (95% CI)^a^ | P-value |
|  | 2.02  (1.99, 2.05) | 2.06  (2.01, 2.10) | 1.75  (1.61, 1.89) | 1.72  (1.56, 1.87) | -0.07 mg  (-0.28, 0.14) | 0.500 |
| Results obtained from linear regression models adjusted for patient age, sex, race, ethnicity, American Society of Anesthesiologists physical status classification, body mass index, comorbidities (dementia, anxiety, cerebrovascular disease, cardiovascular disease, hypertension, chronic obstructive pulmonary disease, kidney disease, diabetes, peripheral vascular disease),use of volatile general anesthetics (e.g., Sevoflurane, Isoflurane), case duration, and surgical department performing procedure. ^a^ Intervention effect is the difference-in-difference-estimate derived from the model. Itis the coefficient of the interaction term between time period (pre versus post-intervention) and exposure included in the regression; ^b^ Includes patients who received at least one dose of midazolam intraoperatively. CI (confidence interval); p.p. (percentage point) | | | | | | |

**A B**


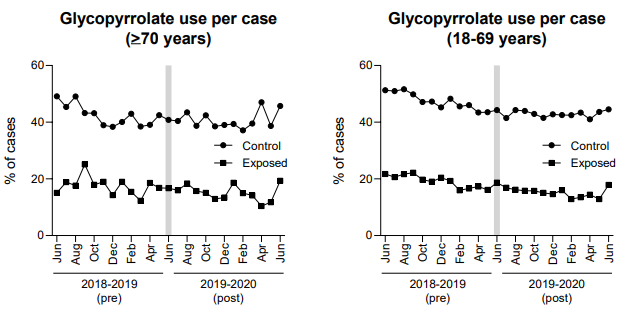


**Supplementary Figure 5: Monthly glycopyrrolate administration during study period**

*Raw trends in perioperative glycopyrrolate use during study period. Percentage of cases in which any glycopyrrolate was administered to patients age (A) 70 years and older (n=20,347 cases) and (B) 18 through 69 years old (n=76,735 cases). Each data point represents (number of cases using glycopyrrolate / total number of cases) calculated by month. Control group represents aggregate data from unexposed hospitals (the comparator arm). Exposed group represents data from intervention site. Grey bar over June 2019 represents the implementation period.*

**Supplementary Table 11: Adjusted glycopyrrolate administration rates before and after alert implementation (patient age 70 years and older)**

| **Outcome** | **Control Facilities (95% CI)** | | **Exposed Facility (95% CI)** | |  |  |
| --- | --- | --- | --- | --- | --- | --- |
| Percentage receiving any glycopyrrolate | Pre-intervention  (N = 6,366) | Post-intervention  (N = 5,991) | Pre-intervention  (N = 4,240) | Post-intervention  (N = 3,750) | Intervention effect  (95% CI)^a^ | P-value |
|  | 42.0%  (40.8, 43.2%) | 40.4%  (39.1, 41.6%) | 18.0%  (16.8, 19.2%) | 16.0%  (14.8, 17.2%) | -0.4 p.p.  (-2.7, 2.0) | p=0.745 |

Results obtained from linear regression models adjusted for patient age, sex, race, ethnicity, American Society of Anesthesiologists physical status classification, body mass index, comorbidities (dementia, anxiety, cerebrovascular disease, cardiovascular disease, hypertension, chronic obstructive pulmonary disease, kidney disease, diabetes, peripheral vascular disease),use of volatile general anesthetics (e.g., Sevoflurane, Isoflurane), case duration, and surgical department performing procedure. ^a^ Intervention effect is the difference-in-difference-estimate derived from the model. It is the coefficient of the interaction term between time period (pre versus post-intervention) and exposure included in the regression. CI (confidence interval); p.p. (percentage point).

**Supplementary Table 12: Adjusted glycopyrrolate administration rates before and after alert implementation (age 18 through 69 years old)**

| **Outcome** | **Control Facilities (95% CI)** | | **Exposed Facility (95% CI)** | |  |  |
| --- | --- | --- | --- | --- | --- | --- |
| Percentage receiving any glycopyrrolate | Pre-intervention  (N = 24,157) | Post-intervention  (N = 22,056) | Pre-intervention  (N = 16,457) | Post-intervention  (N = 14,065) | Intervention effect  (95% CI)^a^ | P-value |
|  | 48.0%  (47.3, 48.7%) | 43.6%  (42.9, 44.3%) | 18.4%  (17.7, 19.0%) | 14.3%  (13.6, 15.0%) | 0.4 p.p.  (-0.9, 1.6) | p=0.551 |

Results obtained from linear regression models adjusted for patient age, sex, race, ethnicity, American Society of Anesthesiologists physical status classification, body mass index, comorbidities (dementia, anxiety, cerebrovascular disease, cardiovascular disease, hypertension, chronic obstructive pulmonary disease, kidney disease, diabetes, peripheral vascular disease),use of volatile general anesthetics (e.g., Sevoflurane, Isoflurane), case duration, and surgical department performing procedure. ^a^ Intervention effect is the difference-in-difference-estimate derived from the model. It is the coefficient of the interaction term between time period (pre versus post- intervention) and exposure included in the regression. CI (confidence interval); p.p. (percentage poin
